# Locating helicopter ambulance bases in Iceland: efficient and fair solutions

**DOI:** 10.1186/s13049-023-01114-9

**Published:** 2023-11-01

**Authors:** Björn Gunnarsson, Kristrún María Björnsdóttir, Sveinbjörn Dúason, Ármann Ingólfsson

**Affiliations:** 1 Institute of Health Science Research, University of Akureyri, Akureyri, Iceland; 2 Akureyri Hospital, Akureyri, Iceland; 3 Alberta School of Business, University of Alberta, Edmonton, Alberta Canada

**Keywords:** Air ambulance, Facility location problem, Fairness

## Abstract

**Background:**

Fixed-wing air ambulances play an important role in healthcare in rural Iceland. More frequent use of helicopter ambulances has been suggested to shorten response times and increase equity in access to advanced emergency care. In finding optimal base locations, the objective is often efficiency—maximizing the number of individuals who can be reached within a given time. This approach benefits people in densely populated areas more than people living in remote areas and the solution is not necessarily fair. This study aimed to find efficient and fair helicopter ambulance base locations in Iceland.

**Methods:**

We used high-resolution population and incident location data to estimate the service demand for helicopter ambulances, with possible base locations limited to twenty-one airports and landing strips around the country. Base locations were estimated using both the maximal covering location problem (MCLP) optimization model, which aimed for maximal coverage of demand, and the fringe sensitive location problem (FSLP) model, which also considered uncovered demand (i.e., beyond the response time threshold). We explored the percentage of the population and incidents covered by one to three helicopter bases within 45-, 60-, and 75-min response time thresholds, conditioned or not, on the single existing base located at Reykjavík Airport. This resulted in a total of eighteen combinations of conditions for each model. The models were implemented in R and solved using Gurobi.

**Results:**

Model solutions for base locations differed between the demand datasets for two out of eighteen combinations, both with the lowest service standard. Base locations differed between the MCLP and FSLP models for one combination involving a single base, and for two combinations involving two bases. Three bases covered all or almost all demand with longer response time thresholds, and the models differed in four of six combinations. The two helicopter ambulance bases can possibly obtain 97% coverage within 60 min, with bases in Húsafell and Grímsstaðir. Bases at Reykjavík Airport and Akureyri would cover 94.2%, whereas bases at Reykjavík Airport and Egilsstaðir would cover 88.5% of demand.

**Conclusion:**

An efficient and fair solution would be to locate bases at Reykjavík Airport and in Akureyri or Egilsstaðir.

**Supplementary Information:**

The online version contains supplementary material available at 10.1186/s13049-023-01114-9.

## Background

Iceland is an island located in the North Atlantic Ocean, in the middle of a storm track that causes strong winds and frequent precipitation [1]. The landmass covers an area of 103,000 km^2^, extending 350 km from north to south and 500 km from east to west. Iceland is the most sparsely populated country in Europe, with only 387,750 inhabitants as of January 1, 2023, and an average population density of 3.76 people/km^2^. The population is unevenly distributed, with most people residing in the southwest part of the country where the capital Reykjavík is located. No one lives in the interior, which is characterized by glaciers, mountains, sand, and lava fields.

Hospital services are also unevenly distributed. A 700-bed national university hospital is located in Reykjavík. There is a 110-bed hospital with some medical and surgical subspecialities in Akureyri and three small hospitals, in Akranes, Ísafjörður, and Neskaupsstaður, that are capable of providing some emergency surgeries under anesthesia (e.g., appendectomy and cesarean section). Given this, providing quality emergency medical services to those in need is challenging. There is a single national emergency dispatch call center for the whole country. Incoming calls are classified by the emergency dispatcher, with the help of a computer program, into one of four priority groups. The highest priority is used for all life-threatening conditions, at the discretion of the dispatcher.

The country has recently become a major tourist attraction, with an estimated 2,400,000 tourists visiting the country in 2023. Many tourists travel by car and are more likely to be involved in traffic accidents than locals [2] who are more experienced in operating vehicles on gravel roads, passing single-lane bridges, and driving over mountain passes that can have ice and snow in any month of the year. Data collected by the Transportation Authority shows that 20.1 tourists per million died or sustained severe injuries in traffic-related accidents during the period of 2013–2022, and more than one-fourth of drivers injured in accidents during the peak years of tourism, 2017–2019, were either tourists or immigrants [3].

Survival probability for out-of-hospital emergencies is an indicator of the quality of care. Research has shown that factors determining survival include the response time of emergency medical services [4] and transport time to a hospital capable of managing emergencies appropriately [5, 6]. In many areas of Iceland, air ambulance services are the only means for rapid retrieval and transport of seriously ill and injured patients to advanced emergency care centers. Most people are flown to the national university hospital by fixed-wing air ambulances based in Akureyri, which transport approximately 200 patients with life-threatening illnesses or injuries per year [7]. Our recent study showed that the median response time for such transports is 84 min [7], during which local healthcare providers are compelled to manage ill and injured patients using limited means. The physician-manned Icelandic Coast Guard Search and Rescue helicopters based at Reykjavík Airport are used for some scene responses as well as secondary transfers—approximately 100–150 patient transports per year. However, the Coast Guard rarely responds to emergencies in the north and east parts of the country that are far away from the base in Reykjavík [8]. There are no other air ambulance bases in the country.

In Norway, one of Iceland’s closest neighbors, the importance of timely response to emergencies is stressed by the government’s official service standards (distance or time from facility); a physician-manned ambulance should be able to reach 90% of the population within 45 min [9]. The ability of helicopter ambulances to respond to scenes and access areas that are difficult to reach by other means helps the country to reach such goals [10]. Seriously ill and injured patients may benefit from timely decision making and procedures performed by highly trained physicians on board [11], as well as rapid transport to an appropriate hospital. The Icelandic authorities have realized this and intend to locate the country’s first helicopter emergency medical service base somewhere in the southwest, but details have not been provided publicly [12]. An official service standard for air ambulance service in Iceland has yet to be set. The Icelandic standard may need to be less stringent than the Norwegian one because of Iceland’s lower population density. The location of helicopter ambulance bases will determine who in the population can or cannot be reached within the set service standard.

A frequently used model for siting ambulance bases is the robust maximal covering location problem (MCLP) [13–17]. The MCLP model aims to maximize coverage of demand within the set service standard (distance or time from base), and the optimal model solution inevitably favors those living in densely populated areas. The model ignores the distance/time for demand beyond the service standard, which can disadvantage people living in the most rural and remote places. Recent research has questioned whether the most efficient use of ambulance resources is fair [18]. The concept of fairness can be included in location problems, and the outcome of such models will likely site bases slightly further away from densely populated areas, which may well introduce additional logistical challenges [16]. The fringe sensitive location problem (FSLP) [19, 20] model provides a relatively simple means to incorporate fairness by optimizing a weighted sum of coverage and distance for those who are not covered within a set service standard [16]. Another means to incorporate fairness is to exclude demand that might be covered using physician-staffed rapid response car, which is faster than helicopter ambulance within variable distance or time [21]. The aim of this study was to use the MCLP and FSLP models to find optimal and fair locations for helicopter ambulance bases in Iceland.

## Methods

### Data material

We used aggregated population and incident data as a proxy for possible demand for helicopter ambulances. Statistics Iceland produced data on a fine grid with 1 km^2^ cells to provide detailed information about the population density of Iceland on January 1, 2022. The National Emergency Number Service provided and gave permission to use incident data for the period from December 2015 to August 2022 (93 months). Ambulances responded to a total of 45,394 highest-priority incidents during this period. Of this total, road ambulances responded to 43,983 incidents, fixed-wing air ambulances to 1216 incidents, and Coast Guard helicopters to 195 incidents. The data included exact locations (latitude and longitude) of each incident, which were aggregated to the same fine grid cells as population data for de-identification. Relative population and incident density in each 1 km^2^ cell were compared using Pearson’s correlation coefficient to measure the linear correlation between the two measures of demand.

We applied to the National Bioethics Committee for study permission (referral no. 22–125), which determined that ethics approval was not required.

### Service standard—response time

The service standard is the desired maximal travel distance or time from the facility to the demand location. In the context of this study, it is the time from an emergency call until the moment of helicopter arrival, referred to as the response time. It consists of a reaction time, which includes the essential preparations for a flight, followed by the flight time. There is no literature on helicopter ambulance reaction times in Iceland. In our models, we used a 15-min reaction time, which is the maximum allowed time for helicopter ambulances in Norway [22], followed by the desired maximal flight time of 30-, 45-, and 60-min. In the mathematical models, we used an average helicopter speed of 220 km/h, a number used in similar studies [14, 15].

### Models

As a proxy for demand for ambulance helicopters, we used, in turn, all populated cells and all cells containing incidents. It was not realistic to use all the demand locations as potential base locations. Instead, we used all 15 airports in Iceland that are used by the fixed-wing ambulance service and six additional airstrips, for a total of 21 potential bases that span all inhabited parts of the country, but exclude the interior.

The optimal base location was determined by applying the MCLP model that maximizes demand and is covered by at least one helicopter ambulance base within the desired response time. The model does this by weighing demand (i.e., cells with more numbers are more likely to be covered than cells with fewer numbers). The model implicitly assumes that each base has an available helicopter at all times. We explored this for 45-, 60-, and 75-min response times and one, two, or three potential helicopter bases.

We addressed fairness in ambulance helicopter base locations using the FSLP. This model has two objectives: to maximize coverage of demand (efficiency) and minimize the weighted response time for those not covered within the service standard (fairness). It does not require a solution that maximizes coverage, but one of the best ways to fulfill the second objective is to cover as much demand as possible within the service standard [20]. The weights for the two objectives were set to 1 and 1000, respectively, to emphasize the fairness objective. The basic idea is to provide some degree of equity by locating facilities closer to demand that is not currently covered. In Additional file 2, we provide details about the FSLP model.

Using both population and incident data, we first modeled base locations conditioned on the existing base at Reykjavík Airport, which is referred to as brownfield analysis. We also computed the optimal and fair base locations assuming no bases existed, which is referred to as greenfield analysis. This resulted in a total of eighteen combinations of conditions for each model. It may be irrational to use helicopters when a physician-staffed rapid response car can respond faster. We therefore repeated the analysis with 18,722 (41.24%) incidents that were greater than 10 km straight-line distance and 15,816 (34.84%) incidents that were greater than 30 km straight-line distance from Landspitali—the national university hospital of Iceland. Finally, we repeated the brownfield analysis with 26,258 (58.5%) incidents that occurred prior to the onset of the COVID-19 pandemic in March 2020, as the pandemic likely affected the distribution of calls for assistance. The models were implemented in R and solved using Gurobi [23].

## Results

**Fig. 1:**
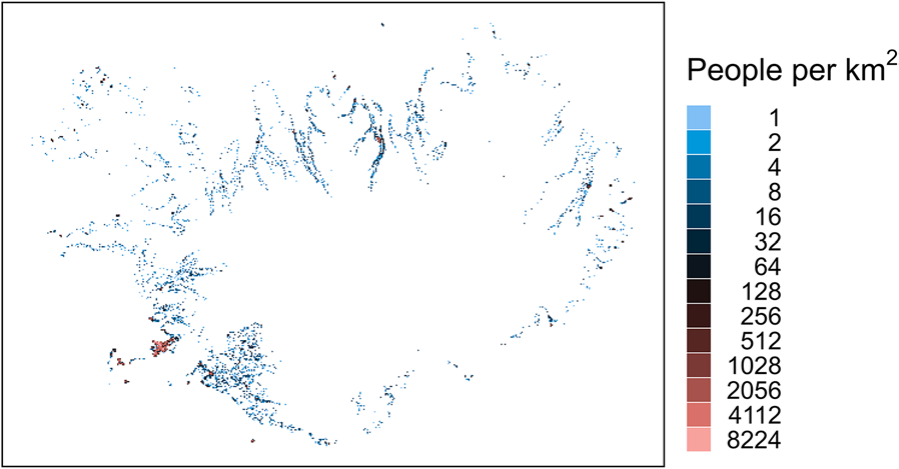
Population density of Iceland as of 2022 in 1 km^2^ cells

**Fig. 2:**
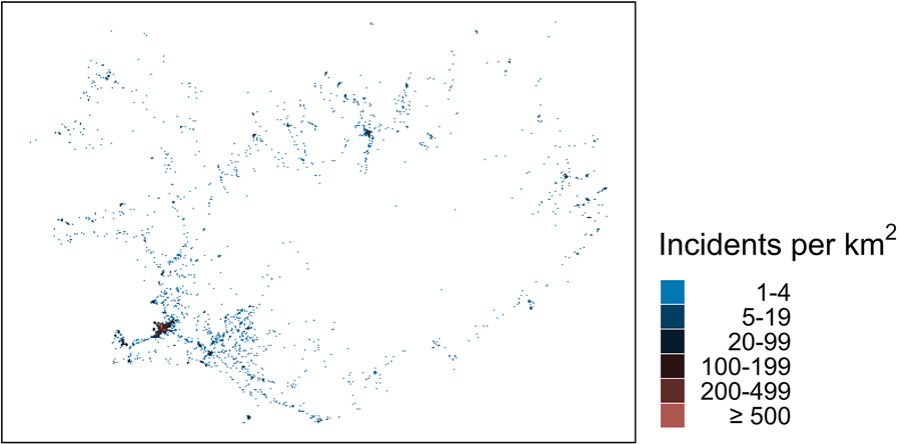
Highest priority incident density in Iceland for December 2015–August 2022 in 1 km^2^ cells

**Fig. 3:**
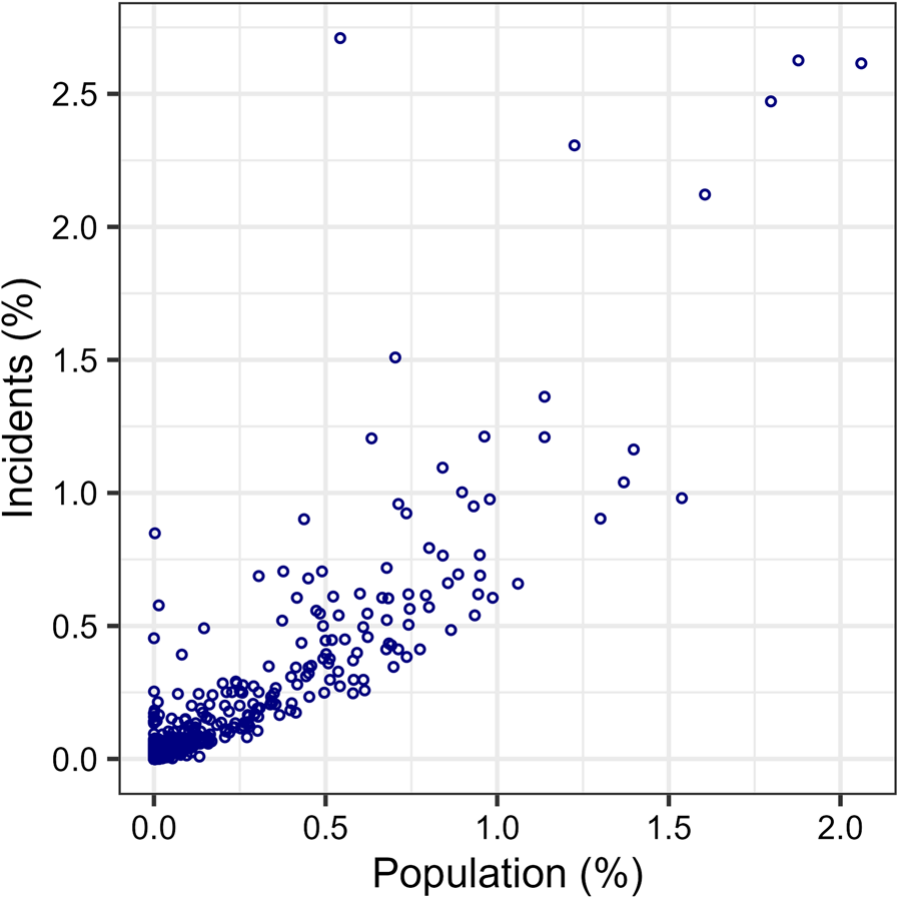
Correlation between the two sets of demand data

**Fig. 4:**
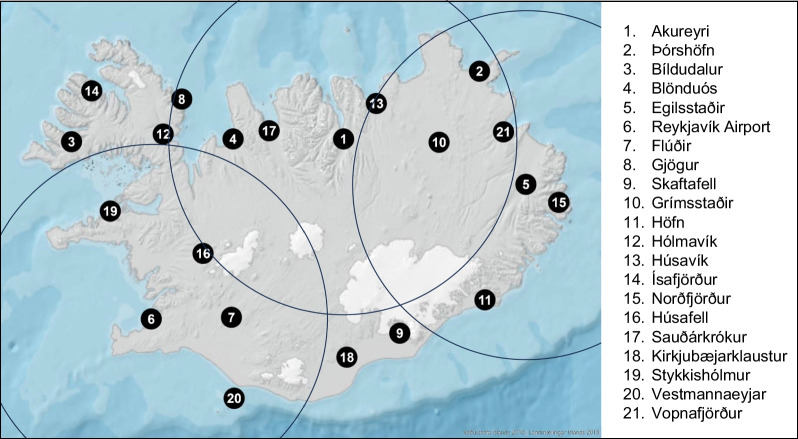
Possible sites for base selection by the location models. The large circles show the area that can be covered within 60 min from bases at Reykjavík Airport, Akureyri and Egilsstaðir. The background image was reprinted from https://geo.vedur.is with permission from Veðurstofa Íslands

Figure 1 shows the population density of Iceland in 1 km^2^ cells. On January 1, 2022, the population was 376,230, and only 3710 of the cells were inhabited. The number of people in the inhabited cells was heavily skewed, with a median (5–95 percentile) of 4 (1–392). Figure 2 shows the incident density (heat map) in the 1 km^2^ cells. Only 2145 cells had one or more of the highest priority incidents. This was also heavily skewed, with a median (5–95 percentile) of 2 (1–112). Figure 3 shows the correlation between the two sets of data. The Pearson’s correlation coefficient was 0.89. Figure 4 shows a map of Iceland with possible base locations randomly numbered. These base numbers are used in Tables 1 and 2.

**Table 1:**
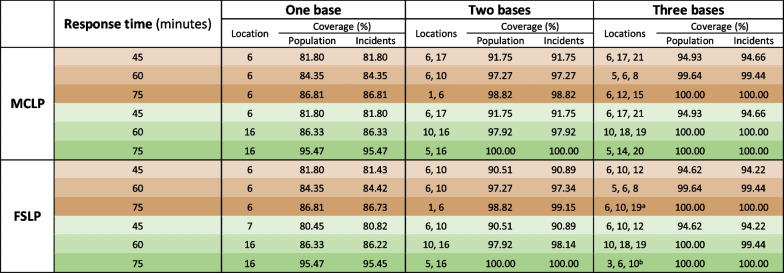
Location of bases for combinations of one, two, and three bases and 45-, 60- and 75-min response times in brownfield (brown cells) and greenfield (green cells) scenarios for the MCLP and FSLP models. Coverage of population and incidents is shown in percentages

The table shows base locations for the incident data. Using population data resulted in different base locations for two combinations: ^a^6, 11, 17 and ^b^4, 5, 6

**Table 2:**
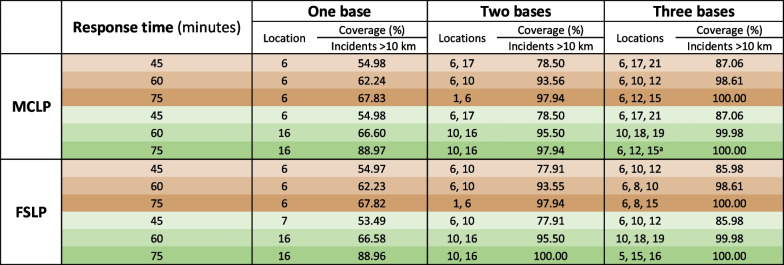
Location of bases for combinations of one, two, and three bases and 45-, 60-, and 75-min response times in brownfield (brown cells) and greenfield (green cells) scenarios, showing coverage for incidents that are greater than a 10 km straight-line distance from the University Hospital of Iceland

Using data for incidents that are greater than a 30 km straight-line distance from the hospital resulted in different base locations for one combination: ^a^5, 14, 20

Optimal solutions for incident data are shown in Table 1, displaying coverage percentage for each combination: one, two, or three helicopter bases; 45-, 60-, or 75-min response time; and greenfield or brownfield scenario. The solutions for population coverage percentage were identical to those shown in Table 1, with two exceptions shown in the footnotes. Six of 21 potential bases were never used. Base locations differed between brownfield and greenfield scenarios in thirteen of the eighteen combinations.

Base locations differed for one combination between the MCLP and FSLP models when siting one base with 45-min response time in a greenfield scenario; the MCLP model located the base at Reykjavík Airport, but the FSLP model sited the base further east, in Flúðir. Base locations differed for one combination in each scenario between the models when two bases were sited at 45-min response time; the MCLP model located the second base in Sauðárkrókur, but the FSLP model located it further east, in Grímsstaðir. Three bases covered all or almost all demand with longer response times, and base locations differed for four of six combinations.

**Fig. 5:**
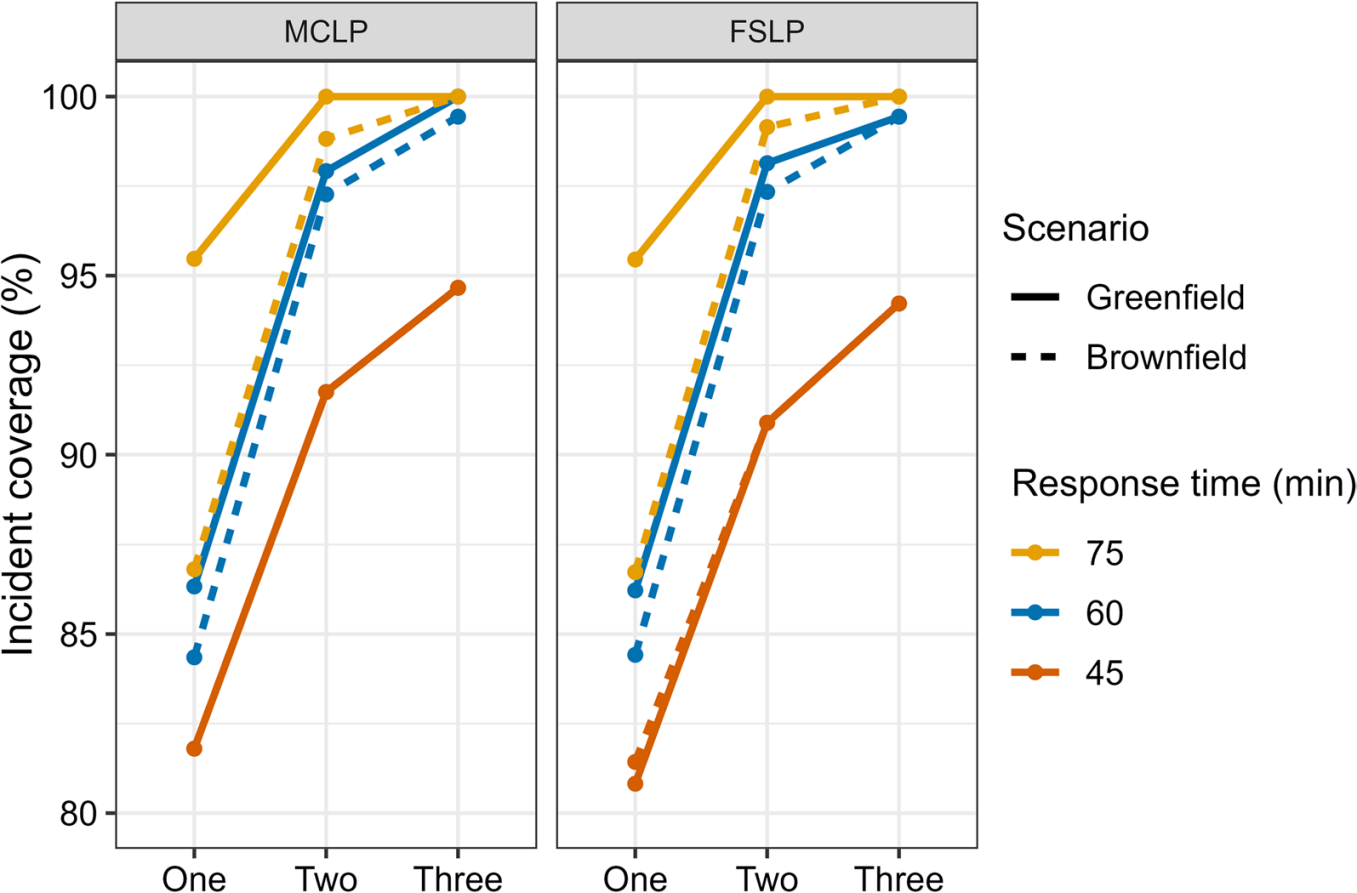
Coverage of incidents for combinations of one, two, and three bases and 45-, 60-, and 75-min response times in brownfield and greenfield scenarios for the MCLP and FSLP models

With only one helicopter ambulance base, it would not be possible to obtain 90% coverage within a 75-min response time (lowest service standard) unless the base was moved from Reykjavík Airport to Húsafell. Adding a base at Egilsstaðir would increase coverage to 100% within 75 min. For a 60-min service standard, 97% coverage is possible with two bases located in Húsafell and Grímsstaðir. This same response time in a brownfield scenario with the second base located at Egilsstaðir would suffice to cover 88.48% of incidents, and if a second base were located in Akureyri, the coverage would be 94.16%. Figure 5 shows the effect of moving or adding bases on incident coverage for different response times.

Solutions for incident data that are greater than a 10 km straight-line distance from the University Hospital of Iceland are shown in Table 2. Base locations differed from those in Table 1 for five of 12 combinations when three bases were sited, and the coverage percentage was lower—particularly when only one base was sited. Base solutions for incident data that are greater than a 30 km straight-line distance from the hospital were identical for all but one combination (see Table 2 footnote). Solutions for incident data prior to the onset of the COVID-19 pandemic are shown in Additional file 2. Base solutions differed from those in Table 1 for half of the combinations when three bases were sited, but coverage percentage was almost identical.

## Discussion

The most important finding of this study is that 97% coverage of estimated demand for out-of-hospital emergencies in Iceland is possible with the addition of one helicopter ambulance base. This is true for a 60-min response time in which the first 15 min are used for crew scrambling and flight preparation. Adding a third base would add little to the coverage.

It is very expensive to open and run helicopter bases and therefore unlikely that more than two helicopter ambulance bases will be operated in Iceland in the foreseeable future. According to both models used, the optimal location of the two bases would be Húsafell in the southwest and Grímsstaðir in the northeast. However, many factors must be considered in the selection of bases for helicopter ambulances. It is expensive to relocate an established base and more expensive to run a base when the staff has to travel long distances from home to work and remain there for days. It is also important to have sufficient access to different services, for example specialized maintenance. It is therefore almost certain that the base at Reykjavík Airport will not be moved.

Grímsstaðir is a farm settlement beside the main road between Akureyri and Egilsstaðir. Locating a second base in Grímsstaðir is infeasible for many reasons (e.g., lack of necessary infrastructure, lack of nearby services, and a very harsh climate). Akureyri and Egilsstaðir both have international airports with the possibility for instrument approaches, which is important considering the climate in Iceland. Akureyri is a town of 20,000 residents with a hospital and necessary infrastructure and services. Locating a new base there would increase coverage for large areas that are underserved today. However, locating a base in Egilsstaðir would likely be favorable to people in the eastern part of the country, where the availability of advanced emergency care is limited.

One strength of this study is the quality of data regarding population and incident locations. Few, if any, studies have used data at this level of granularity for an entire country for the purpose of locating ambulance helicopter bases. It cannot be taken for granted that population density mirrors demand for ambulances, as injuries and illnesses often take place far from home [15] and many incidents involve tourists who do not reside in Iceland. Furthermore, demand can vary between regions (e.g., due to differences in age distribution that have an impact on the likelihood of an emergency) [24]. A study from Norway concluded that it is better to use incident data than population data [15], but our study showed only minor differences, which were limited to the lowest service standard. It is possible, however, that our results could have been impacted by the low frequency of tourist-related emergencies during the COVID-19 pandemic, which overlapped, in part, with the historic incident data. We accounted for this by modeling incident data that was dated prior to the onset of COVID-19 in a brownfield scenario and found that solutions were the same as those based on data for all incidents, except when three bases were sited and coverage was complete or almost complete—as several base solutions are almost equivalent given such conditions. Another potential limitation is the fact that requests for helicopter assistance frequently go directly to the Coast Guard Operations Centre and are not recorded by the national emergency number. Such requests may involve patient transport with the highest priority, but we were unable to obtain accurate data about such missions and acknowledge that this may have influenced our findings.

Most of the literature on ambulance locations has examined how to efficiently allocate resources, and the most widely used measure for efficiency is expected coverage [25]. However, an overarching goal of Icelandic air ambulance services must be to increase equity or fairness in access to advanced healthcare for all. Equity has been evaluated in models for ambulance coverage [18, 26, 27], but there is no consensus on the best way to do this [27]. To our knowledge, the FSLP model has not been used to locate helicopter ambulance bases. The solutions from this model differed from the MCLP model for seven of the eighteen combinations, in some instances with significant consequences for those living in remote and rural places. This was perhaps most obvious when the second of two bases was located in Sauðárkrókur by the MCLP model—leaving the whole eastern part of the country largely uncovered. We find it a major advantage that the model objectives are easily explained and do not require interpretation or special knowledge to understand [18]. Maximum efficiency is not fair to people in remote and rural parts of Iceland. We found the FSLP model to be superior to the MCLP model for locating facilities in our sparsely populated country, even though the models concurred in the likely scenario of two bases and 60-min response time.

Modeling efficient and fair locations for ambulance helicopter bases is by no means a simple task, and methods that are appropriate for locating other services, such as road ambulances or automatic external defibrillators, might not be ideal for this purpose. The coverage construct is based on the notion that the benefit of service is of some positive value [16]. In contrast, the benefit of helicopter ambulance response, as opposed to ground ambulance response, starts some time or distance away from major hospitals, depending on weather, traffic, and other factors [21]. Some helicopter ambulance services even use rapid response cars when cars are deemed to be superior to helicopters or when weather conditions prohibit flying [28–30]. The relative benefit of helicopters over ground ambulances beyond this distance must be some function of distance or time, and the transport time to a hospital clearly impacts patient outcomes. This is, for example, true for patients with time-critical conditions, such as ischemic stroke, for which the benefit of thrombolysis given at a hospital rapidly declines with time [31]. We consider it a potential weakness of both models used in our study that demand close to hospitals has equal weight as demand further away from hospitals. We addressed this by re-running the models after the exclusion of all incidents within a 10 km and a 30 km straight-line distance from the national university hospital in Reykjavík and found that neither changed the outcome when one or two bases were sited. However, this clearly showed that coverage from the existing base is poor.

## Conclusion

An efficient and fair solution is to locate helicopter ambulance bases at Reykjavík Airport and in Akureyri or Egilsstaðir.

### Supplementary Information

**Additional file 1:** Formulation for the FSLP.

**Additional file 2: Table 3.** Location of bases for combinations of one, two, and three bases and 45-, 60-, and 75-minute response times in the brownfield scenario showing coverage of incidents prior to the COVID-19 pandemic.
