# Supplementary material for: Locating helicopter ambulance bases in Iceland: efficient and fair solutions
Source: Scand J Trauma Resusc Emerg Med. 2023 Nov 1;31:70. doi: 10.1186/s13049-023-01114-9 (PMC10621180; doi:10.1186/s13049-023-01114-9)
Supplement: Supplementary file 1 — Additional file 1: Formulation for the FSLP. [file 13049_2023_1114_MOESM1_ESM.pdf]

Additional file 1. Formulation for the FSLP.

In this document, we formulate the fringe sensitive location problem (FSLP), which extends the MCLP to minimize the total distance for uncovered demand. Our formulation is adapted from formulation P3 on pg. 43 in the original paper[1], with some changes to notation and set definitions. In particular, we have written the formulation to avoid explicitly using the sets N_i, in order to make the algebraic formulation closer to the ompr[2] formulation in R.

Sets and indices:

$$\begin{aligned} i \in I &= \text{set of demand nodes} \\ j \in J &= \text{set of potential facility sites} \\ N_i &= \{j : d_{ij} \leq S\} \end{aligned}$$

Parameters:

$$\begin{aligned} a_i &= \text{demand at node } i \\ d_{ij} &= \text{distance from node } i \text{ to node } j \\ S &= \text{maximal covering distance} \\ p &= \text{number of facilities to be sited} \\ w &= \text{weight for total distance for uncovered demand objective} \end{aligned}$$

Decision variables:

$x_j = 1$ if a facility is located at j, 0 otherwise

$z_{ij} = 1$ if node i is not covered and j is the nearest open facility to i, 0 otherwise

Objective:

$$\begin{aligned} \text{minimize } & w \sum_{i \in I} \sum_{j : d_{ij} > S} a_i d_{ij} z_{ij} && \text{[total distance for uncovered demand, with weight } w\text{]} \\ & + \sum_{i \in I} \sum_{j : d_{ij} > S} a_i z_{ij} && \text{[total demand not covered]} \end{aligned} \tag{1}$$

The objective function replaces maximization of coverage (as in the MCLP) with minimization of demand that is not covered, in order to make it easier to combine the two objectives. If w is set to zero, then the focus is only on minimizing the total demand that is not covered, i.e., we recover the MCLP. If w is very large, then the focus is (almost) entirely on minimizing the total distance for uncovered demand.

Constraints:

$$\sum_{j : d_{ij} \leq S} x_j + \sum_{j : d_{ij} > S} z_{ij} \geq 1, \text{ for all } i \in I. \tag{2}$$

This constraint ensures that either (1) node i is covered ($\sum_{j : d_{ij} \leq S} x_j \geq 1$) or (2) node i is not covered and it is assigned to its nearest open facility ($\sum_{j : d_{ij} > S} z_{ij} \geq 1$). The minimization of the objective function will ensure that, at optimality, only one of the conditions (1) and (2) is satisfied, and that of the z_{ij} variables in the second sum, only the z_{ij} for which d_{ij} is smallest will equal 1.

$$\sum_{j \in J} x_j = p \qquad \text{[budget constraint, just like in MCLP]} \tag{3}$$

$$z_{ij} \leq x_j \qquad \text{[node } i \text{ can only be assigned to facility } j \text{ if } j \text{ is open]} \tag{4}$$

$$x_j \in \{0, 1\} \qquad \text{for all } j \in J \tag{5}$$

$$z_{ij} \in [0, 1] \qquad \text{for all } i \in I \text{ and } j \in J \tag{6}$$

To illustrate the problem size, suppose we have $|I| = n_{bl} = 25$ potential base locations and $|J| = n_{dp} = 50000$ demand points. Then we will have 25 binary variables (just like in the MCLP) and $25 \cdot 50000 = 1.25$ million continuous variables – compared to 50 thousand continuous variables for the MCLP. If the number of demand points is reduced to 5000 (for example, by aggregating to a 1 km sqare grid system), then the number of continuous variables drops to $25 \cdot 5000 = 125$ thousand, which reduces the computational effort considerably.

In the "Sample Problems Results" section, Church et al[1] vary p from 1 to 6, and for each value of p, solve three problems: (1) MCLP, corresponding to $w = \infty$ for the FS-MCLP formulation, (2) solely minimize total distance for uncovered demand, corresponding to $w = 0$, and (3) a "compromise solution", for which w is set as follows:

$$w = \frac{\text{increase in coverage from solution (2) to solution (1)}}{\text{decrease in total distance for uncovered demand from solution (1) to solution (2)}} \qquad (7)$$

In the sample problem, solutions (1) to (3) are identical, for $p = 3, 5$ and 6. But for the other values of p there is a trade off: reducing the total distance for uncovered demand can only be done at the cost of reducing coverage. Table 3 and Figure 2 illustrate this. Figure 2 also helps one to visualize the rationale for Equation (7).

This approach is analogous to performance measures used in call centres and emergency departments. "Maximize proportion of callers answered within x minutes" (or "patients seen within x minutes") is analogous to maximizing coverage, and it creates an unfortunate incentive, to ignore those who wait more than x minutes, because they don't count towards the performance measure. Alternative performance measures have been proposed, for example the "average excess waiting time"[3], that is, the average amount by which waiting exceeds the average wait. This performance measure is analogous to the total distance for uncovered demand.

References

1. Church, R., Current, J., & Storbeck, J. (1991). A bicriterion maximal covering location formulation which considers the satisfaction of uncovered demand. *Decision Sciences*, 22(1), 38–52.
2. ompr: Optimization modeling package in R. `https://dirkschumacher.github.io/ompr/`
3. Koole, G. (2003). Redefining the service level in call centers. Rapport technique, Department of Stochastics, Vrije Universiteit, Amsterdam.
