# Supplementary material for: Locating helicopter ambulance bases in Iceland: efficient and fair solutions
Source: Scand J Trauma Resusc Emerg Med. 2023 Nov 1;31:70. doi: 10.1186/s13049-023-01114-9 (PMC10621180; doi:10.1186/s13049-023-01114-9)
Supplement: Supplementary file 2 — Additional file 2: Table 3. Location of bases for combinations of one, two, and three bases and 45-, 60-, and 75-minute response times in the brownfield scenario showing coverage of incidents prior to the COVID-19 pandemic. [file 13049_2023_1114_MOESM2_ESM.pdf]

**Additional file 2. Table 3.**

Location of bases for combinations of one, two, and three bases and 45-, 60-, and 75-minute response times in the brownfield scenario showing coverage of incidents prior to the COVID-19 pandemic.

| | Response time (minutes) | One base | | Two bases | | Three bases | |
|---|---|---|---|---|---|---|---|
| | | Location | Coverage of incidents (%) before COVID-19 | Locations | Coverage of incidents (%) before COVID-19 | Locations | Coverage of incidents (%) before COVID-19 |
| MCLP | 45 | 6 | 80.31 | 6, 17 | 90.72 | 6, 17, 21 | 94.25 |
| | 60 | 6 | 83.53 | 6, 10 | 97.21 | 4, 6, 11 | 99.38 |
| | 75 | 6 | 85.94 | 1, 6 | 99.15 | 6, 12, 15 | 100.00 |
| FSLP | 45 | 6 | 80.31 | 6, 10 | 90.34 | 6, 10, 12 | 93.74 |
| | 60 | 6 | 83.53 | 6, 10 | 97.21 | 4, 6, 11 | 99.38 |
| | 75 | 6 | 85.94 | 1, 6 | 99.15 | 6, 8, 15 | 100.00 |
